# Graphene Papers with Tailored Pore Structures Fabricated from Crumpled Graphene Spheres

**DOI:** 10.3390/nano9060815

**Published:** 2019-05-30

**Authors:** Je Kang, TaeGyeong Lim, Myeong Hee Jeong, Ji Won Suk

**Affiliations:** 1 School of Mechanical Engineering, Sungkyunkwan University, Suwon, Gyeonggi-do 16419, Korea; jebabo5147@gmail.com (J.K.); taegyung95@gmail.com (T.L.); jmh6392@naver.com (M.H.J.); 2 SKKU Advanced Institute of Nanotechnology (SAINT), Sungkyunkwan University, Suwon, Gyeonggi-do 16419, Korea

**Keywords:** graphene, powder, paper, porous structure

## Abstract

Graphene papers have great potential for various applications, such as electrodes in energy storage devices, protective coating, and desalination, because of their free-standing structure, flexibility, and chemical tunability. The inner structures of the graphene papers can affect their physical properties and device performance. Here, we investigated a way to fabricate graphene papers from crumpled reduced graphene oxide (rGO) spheres. We found that ultrasonication was useful for tailoring the morphology of the crumpled graphene spheres, resulting in a successful fabrication of graphene papers with tunable inner pore structures. The fabricated graphene papers showed changes in mechanical and electrical properties depending on their pore structures. In addition, the tailored pore structures had an influence on the electrochemical performance of supercapacitors with the fabricated graphene papers as electrode materials. This work demonstrates a facile method to fabricate graphene papers from crumpled rGO powders, as well as a fundamental understanding of the effect of the inner pore structures in mechanical, electrical, and electrochemical characteristics of graphene papers.

## 1. Introduction

The mass production of graphene powders has been enabled by the chemical oxidation of graphite, followed by the exfoliation of graphite oxide into individual graphene oxide (GO) and a reduction of GO with chemical and/or thermal treatments [1]. A large quantity of reduced graphene oxide (rGO) with high electrical conductivity, high surface area, and mechanical robustness has shown its potential uses in industrial applications such as energy-related devices, water purification, and nanocomposites among others [2,3,4,5,6,7].

Among several methods to fabricate macroscopic structures from graphene flakes, a paper-like film presents an attractive form because of its flexibility, mechanical strength, electrical conductivity, and chemical tunability [8,9]. These intriguing characteristics of graphene papers have enabled unique applications, including supercapacitor electrodes, filter membranes, actuators, sensors, and protective coating [10,11,12,13,14]. In general, graphene papers can be fabricated by the vacuum-filtration of GO dispersion in a solvent and subsequent reduction processes of the GO papers [15,16]. This process provides well-stacked layered inner structures in graphene papers. However, some applications require a porous structure in order to achieve a desirable performance. For example, the restacking of graphene flakes leads to limited ion-transport paths in electrodes of supercapacitors based on electrical double layer capacitance (EDLC), hindering the development of high-performance supercapacitors using graphene papers [17,18,19]. Nanoparticles, such as SiO_2_ [20] or polymers [21], have been utilized as templates in order to make porous graphene spheres. In the template-based fabrication, nanoparticles were wrapped with graphene flakes and removed after forming robust three-dimensional (3D) graphene frameworks. More approaches have been developed to control the inner structures of the graphene papers. For example, carbon black particles [22] or carbon nanotubes (CNTs) [23,24] have been used as spacers between graphene layers, generating open nanochannel structures in graphene composite films. In addition, crumpled graphene was developed for obtaining the appropriate pore structures in electrode materials by using aerosol spray drying [19] or the freeze-drying of GO [18]. Subsequently, the crumpled GO powders were reduced in chemical or thermal treatments. However, crumpled shapes hinder the formation of paper-like films because of their reduced contact area among graphene layers and their resulting weak interactions. Therefore, an additional material, such as a binder, is necessary to form paper-like films from the crumpled graphene.

In this work, we investigated a simple method to fabricate a paper-like film from crumpled rGO spheres. In order to tailor the morphology of the crumpled graphene spheres, ultrasonication was utilized, providing successful fabrication of graphene papers with porous inner structures. The mechanical and electrical properties of the fabricated graphene papers were studied according to the sonication time. In addition, supercapacitors with a two-electrode configuration were assembled with the fabricated graphene papers as electrode materials, and their electrochemical performances were evaluated with respect to the sonication treatment.

## 2. Materials and Methods

### 2.1. Tailoring the Morphology of Crumpled Graphene Spheres and Fabrication of Papers

Commercially available rGO powders (TGF600, Grapheneall, Suwon, Korea) were used for this work. The synthesis of the crumpled rGO powders was performed according to the following procedure. First, GO was obtained by the modified Hummer’s method [25] and then partially reduced with L-ascorbic acid in a solution [26]. The partially reduced GO flakes were then spray-dried, forming graphene powders with crumpled spherical morphology [27]. The crumpled powders were additionally reduced by thermal annealing at 1000 °C. 

Experiments started from the commercial, crumpled rGO powders. The shape of the rGO powders was controlled using ultrasonic treatments. The commercial rGO powders were used as received in this work, except the sonication treatments. The as-received rGO powders were mixed with dimethylformamide (DMF) at a concentration of 3 mg/40 mL and the mixture was placed in a bath sonicator with a power of 150 W. Each mixture was treated for different times: 2, 4, 6, 10, 14, and 20 h. Graphene papers were prepared by vacuum-filtration of the sonicated mixture with an anodic aluminum oxide (AAO) membrane filter (0.02 μm pore size, Whatman, Pittsburgh, PA, USA).

### 2.2. Characterization of rGO Powders and Fabricated Graphene Papers

The morphology of the rGO powders was observed by scanning electron microscopy (SEM, JSM-7600, Jeol, Tokyo, Japan) before and after the sonication treatments. The chemical structures of rGO were characterized with X-ray photoelectron spectroscopy (XPS, ESCALAB-250 with a monochromated Al Kα radiation, Thermo-Scientific, Waltham, MA, USA). The C 1s core-level spectra were deconvoluted with Gaussian-Lorentzian functions after the background signal was subtracted by the Shirley-background model. Raman spectroscopy (ALPHA300M with a 532 nm wavelength laser, WiTec, Ulm, Germany) was also used to characterize the rGO powers before and after the sonication treatments.

### 2.3. Mechanical, Electrical, and Electrochemical Measurements of Graphene Papers

The mechanical properties of the fabricated graphene papers were characterized by a universal tensile machine after cutting the paper into rectangular strips [8,16]. In order to evaluate stresses from the measured forces, the thickness of the papers was measured by observing the cross sections of the fractured parts in a SEM. Sheet resistances of the fabricated graphene papers were measured by the van der Pauw method with four-point contacts [16,28]. Electrical conductivities were calculated from the sheet resistances using the measured thicknesses. All the measurements were repeated with more than four samples at one condition.

The electrochemical performance of a supercapacitor was characterized with a symmetric two-electrode cell configuration by following the literature [2,29,30,31]. The supercapacitor test cell consisted of two rGO paper electrodes, two current collectors (conductive polymer film, z-flo 2267p), a porous separator (3501, Celgard, Charlotte, NC, USA), and an aqueous electrolyte [2]. The rGO papers were punched into circular electrodes with a diameter of 1 cm and 6 M KOH solution was used as an aqueous electrolyte. All the components were assembled into a test cell by a fixture with two stainless steel plates [2]. Cyclic voltammetry (CV) and galvanostatic charge/discharge were examined using both potentiostat and galvanostat (Metrohm, Autolab PGSTAT204, Utrecht, Netherlands) [30]. The specific capacitance of a single electrode, *C*_sp_, was calculated from the CV curves by means of the following equation:

(1) $$C_{sp}=\frac{2\oint IdV}{mv\Delta V}$$

where *I* is the voltammetric current, *m* is the mass of a single electrode, *V* is the voltage of the supercapacitor, ∆*V* is the voltage window of the scan, and *ν* is the scan rate.

The specific capacitance of a single electrode was also obtained from the galvanostatic charge/discharge curves by using the following equation:

(2) $$C_{sp}=\frac{2I}{\left(dV/dt\right)m}$$

where *I* is the constant current, *m* is the mass of a single electrode, and d*V*/d*t* was obtained by the slope of the linearly fitted discharge curve.

## 3. Results and Discussion

### 3.1. Tailoring the Morphology of Crumpled Graphene Spheres with Ultrasonication

**Figure 1:**
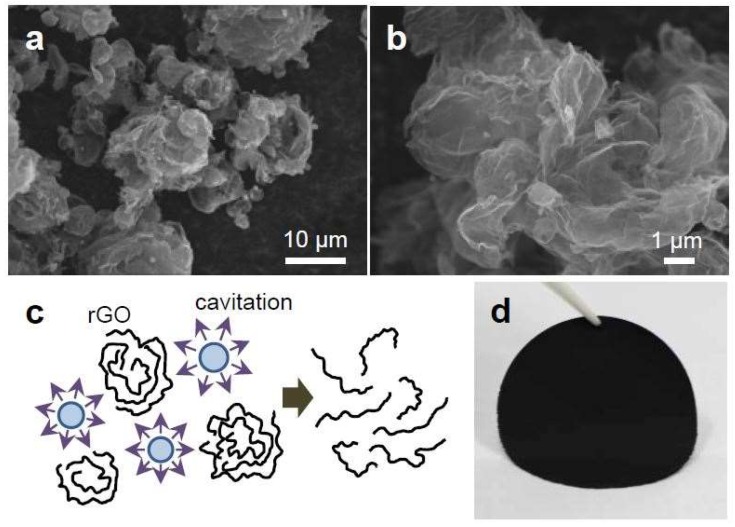
Fabrication of a graphene paper by using ultrasonication treatments on crumpled rGO powders. (**a**,**b**) SEM images of crumped and spherical rGO powders. (**c**) Schematic illustration of sonication treatment on the crumpled rGO powders. (**d**) Optical image of an rGO paper fabricated from the sonicated rGO powders.

The rGO powders had crumpled spherical shapes because a GO dispersion was spray-dried and thermally reduced after partial reduction by L-ascorbic acid in a GO solution. Figure 1a,b show the morphology of the crumpled rGO powders. The crumpled shapes of the rGO powders are useful for several applications; for example, they can be utilized as electrode materials in supercapacitors since the porous structures of the crumpled powders improve the ion diffusion of an electrolyte, resulting in the enhanced electrochemical performance of supercapacitors [19,32]. However, due to the crumpled spherical shapes of the powders, it was not possible to fabricate free-standing papers, which requires additional materials such as binders to form paper-like films from crumpled graphene powders [19]. To tailor the crumpled shapes of the rGO powders and make them flat, we treated the rGO powders dispersed in DMF with ultrasonication. It has been known that the sonication generates cavitations that grow and collapse in a solution, which transforms acoustic energy into mechanical energy with high shear forces [33,34]. We found that this ultrasonication treatment could change the morphology of the crumpled rGO powders (Figure 1c), enabling the fabrication of rGO papers by conventional vacuum filtration as shown in Figure 1d.

**Figure 2:**
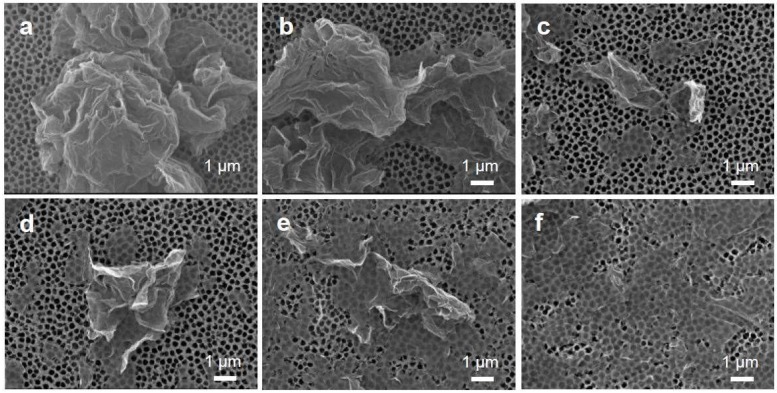
Scanning electron microscopy (SEM) images of rGO flakes placed on anodic aluminum oxide (AAO) membrane filters after sonication treatments for (**a**) 2, (**b**) 4, (**c**) 6, (**d**) 10, (**e**) 14, and (**f**) 20 h.

To observe the change of the morphology of individual rGO powders, a small amount of an rGO solution was dropped on an AAO membrane filter as shown in Figure 2 [35]. The rGO powders with sonication for 2 and 4 h still had crumpled morphology, thus they could not form paper-like films. However, the rGO powders with sonication for more than 6 h had unfolded and flat shapes. The rGO powders with more sonication treatments showed flatter morphology.

**Figure 3:**
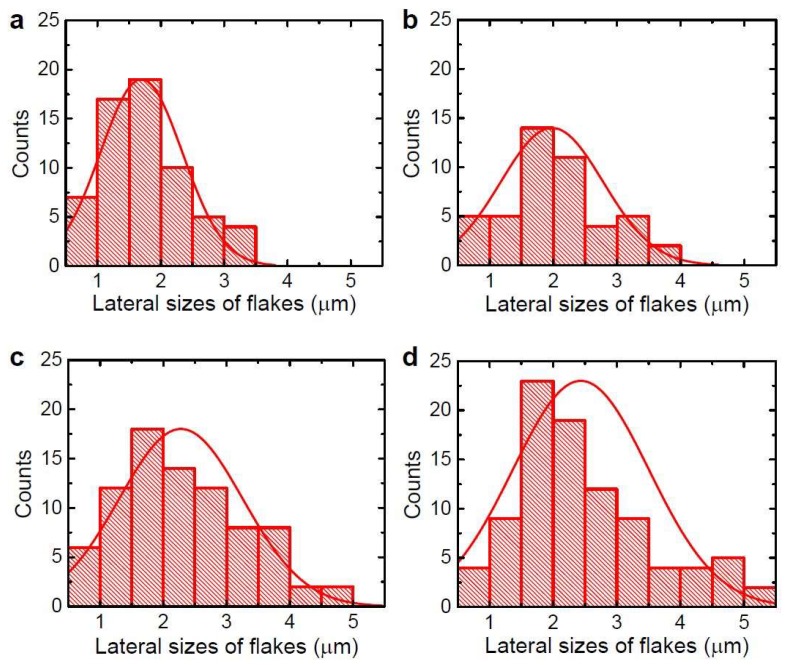
Lateral-size distributions of rGO flakes after sonication treatments for (**a**) 6, (**b**) 10, (**c**) 14, and (**d**) 20 h. The solid lines represent Gaussian fits to the data.

**Figure 4:**
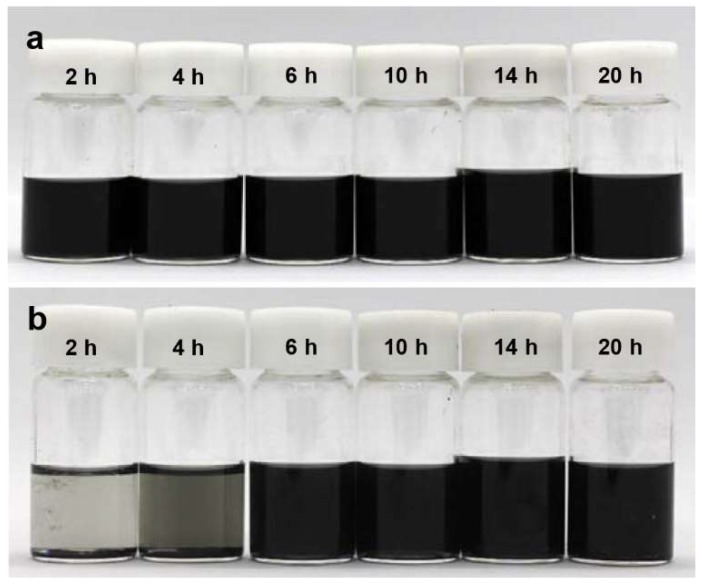
Stability test of sonicated rGO powders dispersed in dimethylformamide (DMF). (**a**) As-prepared rGO dispersions. (**b**) rGO dispersions after 3 months.

The lateral sizes of unfolded rGO flakes were characterized with the observed SEM images. Figure 3 shows histograms of lateral size distributions depending on the sonication time. This indicates that sonication treatment for more than 6 h did not reduced the lateral sizes of the rGO flakes; the average lateral sizes of the flakes were 1.69, 1.98, 2.28, and 2.44 for 6, 10, 14, and 20 h cases, respectively. The unfolded shapes of the crumpled rGO powders affected dispersion stability in DMF. Figure 4a shows rGO mixtures just after sonication treatments. After three months, rGO dispersion in DMF with sonication treatments for 2 and 4 h had significant sediment on the bottom of the bottles while the rGO dispersion with longer sonication treatments showed better dispersion stability as shown in Figure 4b. This confirms that the sonication treatment is an effective way to change the morphology of the crumpled rGO powders.

**Figure 5:**
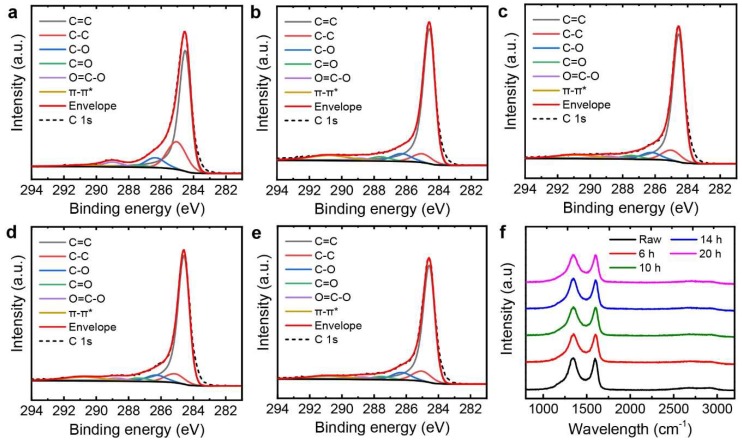
(**a**–**e**) C 1s core-level X-ray photoelectron spectroscopy (XPS) spectra of rGO papers; sonication times are (**a**) 0, (**b**) 6, (**c**) 10, (**d**) 14, and (**e**) 20 h. (**f**) Raman spectra of rGO papers before and after sonication treatments.

XPS was used to further investigate the effect of the sonication treatments on the rGO powders. Figure 5a–e shows the C 1s core-level spectra of the rGO powders with the sonication treatments for 0, 6, 10, 14, and 20 h. The sp^2^-hybridized carbon (C=C) bonding positioned at the binding energy of 284.5 eV was modeled using the asymmetric Doniach-Sunjic line shape [36,37,38,39]. Other spectral components were fitted by Gaussian-Lorentzian product formula corresponding to the sp^3^-hybridized carbon (C–C) at 285.1 eV, C–O at 286.3 eV, C=O at 287.5 eV, and O=C–O at 288.8 eV [40,41]. In addition, the π-π* transition in aromatic systems was located at the binding energy of 290.7 eV [15]. Based on the XPS analysis, the C/O ratios were evaluated for the sonicated rGO powders and showed little variation according to the sonication time; the C/O ratios were 5.67, 5.81, 5.71, and 5.65 for the sonication times of 6, 10, 14, and 20 h, respectively. Therefore, the sonication treatments did not highly affect the chemical structures of the rGO powders.

Raman spectroscopy was also used to characterize the influence of the sonication on the rGO powders. Figure 5f shows typical Raman spectra of rGO powders having strong D and G bands positioned at around 1350 and 1580 cm^−1^, respectively [1]. The integrated intensity ratios of the D band to the G band, *I*_D_/*I*_G_, were 2.29, 2.35, 2.37, and 2.36 for the sonication times of 6, 10, 14, and 20 h, respectively, while the raw powders showed the *I*_D_/*I*_G_ of 2.29. It has been known that *I*_D_/*I*_G_ can indicate the ratio of disordered carbons in graphene nanosheets [42]. Based on the unchanged Raman and XPS spectra after the sonication treatments, we could conclude that the rGO powders kept their chemical and structural states even after long sonication treatments, indicating that the morphology of the rGO powders was only tailored.

**Figure 6:**
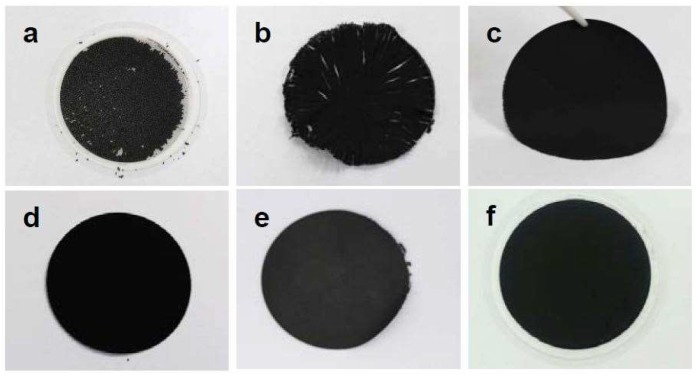
Optical images of the fabricated rGO papers after sonication treatments for (**a**) 2, (**b**) 4, (**c**) 6, (**d**) 10, (**e**) 14, and (**f**) 20 h.

**Figure 7:**
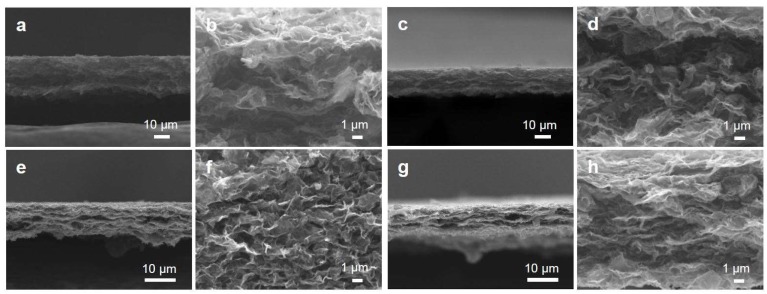
SEM images of cross sections of the fabricated rGO papers after sonication treatments for (**a**,**b**) 6, (**c**,**d**) 10, (**e**,**f**) 14, and (**g**,**h**) 20 h.

The rGO papers were fabricated by conventional vacuum filtration [15,43]. It was found that sonication treatments for more than 6 h enabled the formation of paper-like films and the successful delamination of the paper from the membrane filter without any damage; as shown in Figure 6, sonication treatments for 2 and 4 h were not enough to form continuous graphene papers. The cross sections of the papers were observed to understand the inner structures of the papers. Figure 7 shows the cross-sectional views of the fabricated papers after the sonication treatments for 6, 10, 14, and 20 h. The thicknesses of the papers decrease with an increase in the sonication time; the average thicknesses were 20.9, 16.3, 9.6, and 8.3 μm for 6, 10, 14, and 20 h, respectively. The decrease of the paper thicknesses is attributed to the change of the rGO powder shapes and the paper pore structures; the high-magnification images of the cross sections show that the rGO papers treated for shorter sonication times had large pores while the rGO papers with the sonication treatment for 20 h had a layered, stacking configuration. As discussed with Figure 2, longer sonication treatments generated more unfolded and flatter rGO flakes, which were favorable for making layered paper-like films.

### 3.2. Mechanical, Electrical, and Electrochemical Characteristics of the Graphene Papers

**Figure 8:**
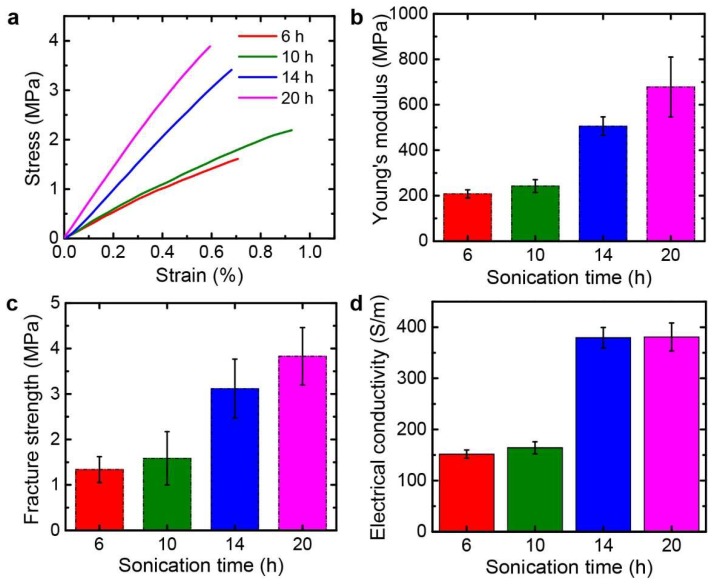
Mechanical and electrical properties of the fabricated rGO papers depending on different sonication times. (**a**) Stress-strain curves. (**b**) Young’s modulus. (**c**) Fracture strength. (**d**) Electrical conductivity.

The rGO papers with tailored pore structures were tested to understand their mechanical, electrical, and electrochemical properties. Mechanical properties were measured by tensile tests after cutting the papers in a rectangular shape. Figure 8a shows representative stress-strain curves for each sample as a function of the sonication time. Better stacking configurations with more layered structures can be obtained by longer sonication treatments, which can generate stronger interactions among rGO flakes due to more contact areas. This leads to the increase of the mechanical properties as the sonication time increases. The Young’s modulus and fracture strength of the rGO papers increased from 208 ± 17.6 and 1.3 ± 0.28 MPa for 6-h sonication to 678 ± 132 and 3.8 ± 0.63 MPa for 20-h sonication as the sonication time increases (Figure 8b,c). Similar to the mechanical properties, the electrical conductivities of the rGO papers were affected by the change of the pore structures of the rGO papers. As shown in Figure 8d, the electrical conductivities increased from 152 ± 8.1 S/m for 6-h sonication to 381 ± 27 S/m for 20-h sonication. Because the rGO papers were prepared with the crumpled rGO powders, the mechanical and electrical properties were lower than conventional rGO papers which were typically obtained using chemical or thermal reduction of GO papers with better layered configurations [16,44]. On the other hand, this work demonstrates an alternative way to fabricate graphene papers directly from crumpled rGO powders. Furthermore, this provides better understanding of tunability of mechanical and electrical properties of the graphene papers with tailored inner pore structures.

**Figure 9:**
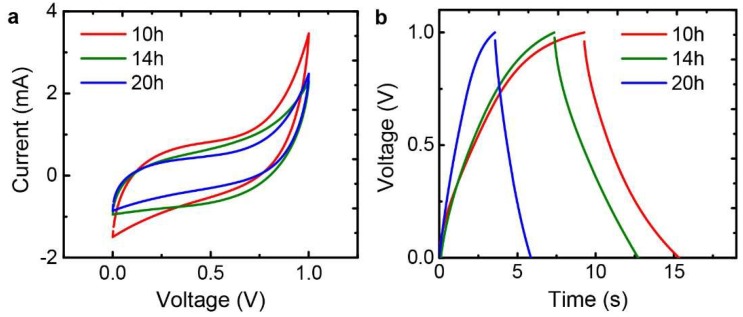
Electrochemical performance of the fabricated rGO papers as electrode materials in supercapacitor cells with 6 M KOH electrolyte depending on different sonication times. (**a**) CV curves at a voltage ramp rate of 100 mV/s. (**b**) Galvanostatic charge/discharge curves at a current density of 2 A/g.

Additionally, electrochemical characteristics of the fabricated rGO papers were examined from supercapacitors based on EDLC. In general, porous structures are desirable for enhancing ion diffusion and adsorption in electrodes of supercapacitors. Since the rGO paper is free-standing, flexible, and electrically conductive, it is a good candidate for the binder-free electrode materials of supercapacitors, especially flexible supercapacitors. Graphene supercapacitors were assembled with two fabricated rGO papers as binder-free electrodes that are isolated by a porous separator. Figure 9 shows the CV and charge/discharge curves of the supercapacitor cells with the rGO paper electrodes. The rGO paper with 10-h sonication shows the largest area in the CV curve, meaning that it has the highest specific capacitance [45]. This is because it allows relatively high ion diffusion rate and ion adsorption area due to its increased porosity compared to other papers. In this respect, the specific capacitance from the CV curves at a voltage ramp rate of 100 mV/s changed as the sonication time varied; 61.1, 48.8, and 38.7 F/g for 10, 14, and 20 h, respectively. Similarly, the charge/discharge curves at a current density of 2 A/g showed the specific capacitances of 56.8, 49.1, and 20.0 F/g for 10, 14, and 20 h, respectively. As a result, we found that the increased pore structures provided higher specific capacitance of the supercapacitor based on EDLC, which provides insight into the development of graphene supercapacitors from crumpled graphene spheres.

## 4. Conclusions

Since graphene powders are commercially available these days, the development of an appropriate method to directly use the graphene powders in a paper-like form is useful for certain applications, such as flexible supercapacitors, actuators, sensors, composites, and water purification. In this respect, this work demonstrates a simple but useful way to make graphene papers directly from crumpled graphene powders by using ultrasonication treatment. The simple sonication treatments tailored the morphology of the crumpled rGO spheres due to the strong cavitation effect, which generated unfolded and flatter rGO flakes. This leads to the successful fabrication of graphene papers directly from the rGO powders. The fabricated graphene papers showed the change of the pore structures depending on the sonication time, resulting in a change of the mechanical and electrical properties of the papers. In addition, supercapacitors with the graphene papers were evaluated as a function of the sonication time. It was found that more pore structures enhanced the specific capacitances of the supercapacitors. This work provides an efficient way to make graphene papers directly from rGO powders and enabled us to better understand the mechanical, electrical, and electrochemical properties of the fabricated graphene papers according to their inner pore structures.
